# Conditioned Medium from Human Adipose-Derived Mesenchymal Stem Cell Culture Prevents UVB-Induced Skin Aging in Human Keratinocytes and Dermal Fibroblasts

**DOI:** 10.3390/ijms21010049

**Published:** 2019-12-19

**Authors:** Lu Li, Hien T.T. Ngo, Eunson Hwang, Xuan Wei, Ying Liu, Jia Liu, Tae-Hoo Yi

**Affiliations:** 1 Shanghai Institute for Advanced Immunochemical Studies, ShanghaiTech University, Shanghai SH021, China; lilu@shanghaitech.edu.cn (L.L.); weixuan@shanghaitech.edu.cn (X.W.); 2 College of Life Sciences, Kyung Hee University, 1732, Deogyeongdae-ro, Giheung-gu, Yongin-si, Gyeonggi-do 17104, Koreafirsthes@khu.ac.kr (E.H.); y.liu0915@khu.ac.kr (Y.L.)

**Keywords:** human adipose-derived mesenchymal stem cells-conditioned medium, UVB radiation, MAPK/AP-1, NF-κB/HO-1, TGF-β/SMAD

## Abstract

Human adipose-derived mesenchymal stem cells-conditioned medium (ADSC-CM) contains cytokines and growth factors that can facilitate the regeneration and repair of various tissues and organs. In the present study, the protective activity of ADSC-CM treatment was investigated in UVB-irradiated human keratinocyte cell line HaCaTs and normal human dermal fibroblasts (NHDFs). It was found that ADSC-CM can modulate the expression of the signaling molecules in the early UVB responsive signaling pathways, including mitogen activated protein kinases (MAPKs), activator protein 1 (AP-1), and nuclear factor kappa B (NF-κB). In addition, ADSC-CM treatment could upregulate antioxidant response element (ARE) such as phase II gene heme oxygenase-1 (HO-1) and increase the expression of collagen synthesis enhancer gene transforming growth factor-β (TGF-β). The expression of matrix metalloproteinase-1 (MMP-1) and procollagen type I synthesis inhibitors such as interleukin-6 (IL-6) was also found to be suppressed upon ADSC-CM treatment. Taken together, our study illustrates the anti-photoaging activities of ADSC-CM in cell-based models.

## 1. Introduction

Skin aging is a complex, progressive biological phenomenon that is associated with chronologic aging and photoaging [1]. Chronologic aging-related reduced response to growth factors, degradation of extracellular matrix, decreased expression of collagen molecules, and disrupted procollagen activity are all observed in photoaging skin, suggesting that chronologic aging and photoaging may share common mechanisms [2]. Development of effective anti-photoaging strategies are important for reducing the risk of ultraviolet B (UVB) radiation.

There are two major signaling pathways involved in the response to UVB radiation. The first is the MAP kinase (MAPK) signal transduction pathways where membrane receptors are activated by UVB-triggered reactive oxygen species (ROS). Phosphorylation of MAPK activates the c-Jun transcription factor as well as nuclear factor kappa B (NF-κB), followed by the activation of transcription factor activation protein 1(AP-1) complex [3]. Both the AP-1 and NF-κB pathways may affect the homeostasis of matrix metalloproteinases (MMPs) and heme oxygenase-1 (HO-1) during skin photoaging. MMPs are zinc-dependent endoproteinases that can degrade collagen and elastic fibers, which may lead to wrinkle formation [4,5]. The second pathway involves UVB-dependent regulation of TGF-β/Smad signaling transduction. TGF-β is an important profibrotic cytokine and functions by binding to specific cell-surface receptors. UVB irradiation modulates the phosphorylation of type II TGF-β receptor and stimulates R-Smads (Smad2, Smad3, and Smad7) activation, which are thought to play pivotal functions in skin damage [6,7].

Compared with bone marrow stem cells, adipose-derived mesenchymal stem cells (ADSC) are approximately 40-fold greater in abundancy and display multi-lineage developmental plasticity with respect to gene profiling [8,9]. Recent studies have illustrated that ADSC can secrete a variety of biologically active molecules that affect the surrounding microenvironment via a paracrine mechanism [10]. These cellular factors render ADSC-derived conditioned medium a valuable source for therapeutic applications [11]. Notably, conditioned medium from ADSC culture (ADSC-CM) can be used as an anti-oxidant agent for skin to reduce the rate of collagen degradation and repair wounds in animal models [12]. In addition, ADSC-CM exhibited antioxidant effects in oxidative injury models on human dermal fibroblasts (HDFs) [13]. Although the wound-healing and antioxidant activities of ADSCs and ADSC-CM are known, their function in the photoaging of skin have not been well characterized. In the present study, we sought to elucidate the molecular mechanism responsible for the photoprotective effects of ADSC-CM using the UVB irradiation model in cultured human keratinocytes and human dermal fibroblasts.

## 2. Results

### 2.1. Intracellular Reactive Oxygen Species (ROS) Production Activity

**Figure 1:**
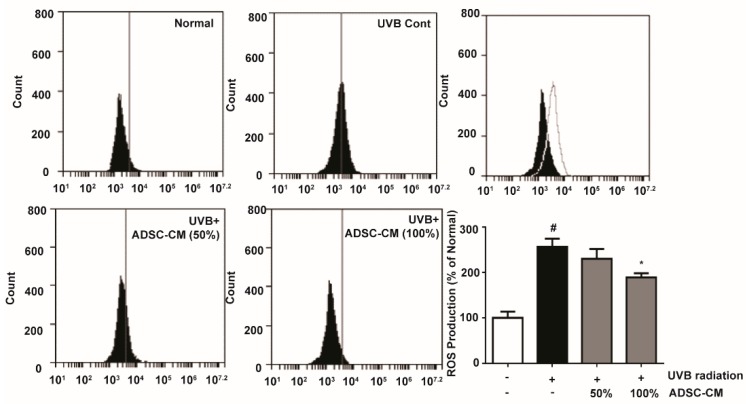
Flow cytometry showing the reactive oxygen species (ROS) levels in HaCaTs, as determined 2′,7′-dichlorodihydrofluorescein diacetate (DCFH-DA) conversion reaction. The number of cells is plotted versus the FL-1 channel for 2′,7′-dichlorofluorescein (DCF) fluorescence. The values in the bar plot are shown as mean ± SD. ^#^ *p* < 0.05, compared to the irradiated, human adipose-derived mesenchymal stem cells-conditioned medium (ADSC-CM) free control. * *p* < 0.05, compared to the UVB-irradiated control.

To determine the production of ROS stimulated by UVB, 2′,7′-dichlorodihydrofluorescein diacetate (DCFH-DA) was applied to immortalized human keratinocyte cells HaCaTs and its oxidation product 2′,7′-dichlorofluorescein (DCF) was measured by flow cytometry. UVB-irradiated cells showed a 156.1% higher ROS generation than non-irradiated cells. Treatment with 50% and 100% ADSC-CM led to a 10.1% and 26.2% decrease in ROS level, respectively, when compared to the non-treated cells (Figure 1).

### 2.2. The Effects of ADSC-CM on the Apoptosis of Normal Human Dermal Fibroblasts (NHDF) cells

**Figure 2:**
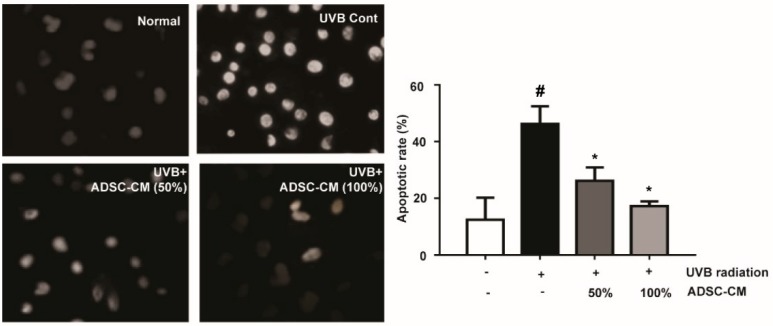
The effects of ADSC-CM on UVB-induced apoptosis of normal human dermal fibroblasts (NHDF) cells, as determined by Hoechst 33258 staining. Magnification scale: 400×. Apoptotic rate was calculated as the percentage of apoptotic cells over the total cell numbers in five randomly selected areas. Values are shown as mean ± SD. ^#^ *p* < 0.05, compared with the non-irradiated control. * *p* < 0.05, compared with the irradiated, ADSC-CM free control.

The results showed that UVB significantly induced typical apoptotic nuclear morphology including shrinkage, nuclei fragmentation, and intense fluorescence due to the damage of the outer membrane (Figure 2). However, cell apoptosis was prevented by the treatment of ADSC-CM. This result clearly suggests that ADSC-CM inhibited UVB-induced apoptosis of NHDF cells.

### 2.3. The Effects of ADSC-CM on MMP-1 and IL-6 Production in Cultured HaCaTs and NHDFs

**Figure 3:**
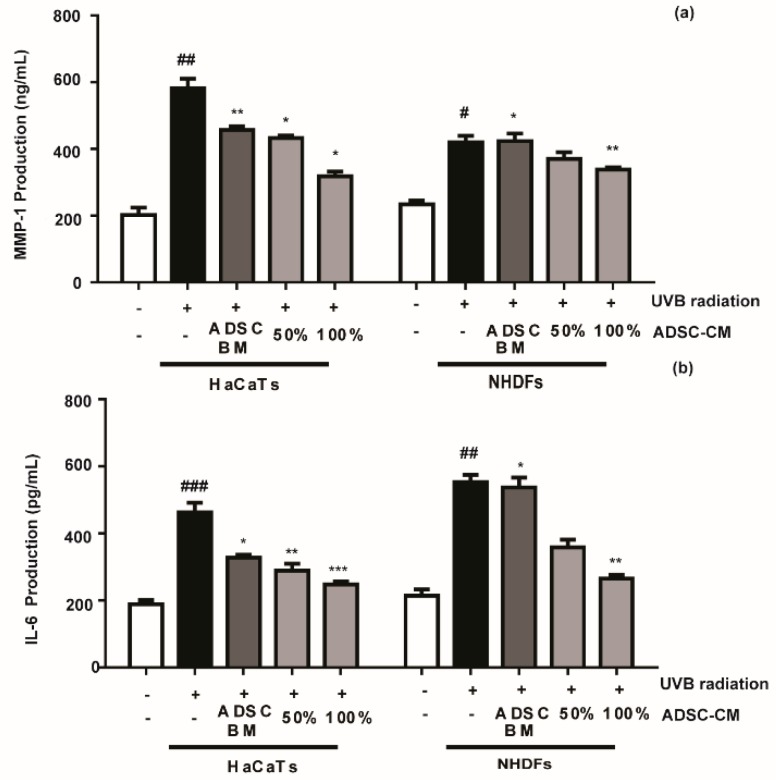
The effects of ADSC basal medium (ADSC-BM) and ADSC-CM on UVB-induced MMP-1 (**a**) and IL-6 (**b**) production. HaCaTs and NHDFs were treated with 100% ADSC-BM or 50% or 100% ADSC-CM, UVB-induced MMP-1 secretion and IL-6 biosynthesis were determined by enzyme-linked immunosorbent assay (ELISA). Values are shown as mean ± SD. ^#^ *p* < 0.05, ^##^ *p* < 0.01, and ^###^ *p* < 0.001, compared with the non-irradiated control. * *p* < 0.05, ** *p* < 0.01, and *** *p* < 0.001, compared with the irradiated, ADSC-CM free control.

Compared with the non-treated control, ADSC-CM treatment decreased the UVB-stimulated secretions of MMP-1 and IL-6 in a dose-dependent manner. The MMP-1 level in HaCaT cells was decreased by 25.8% and 45.5% in the presence of 50% and 100% ADSC-CM, respectively (Figure 3a), while the UVB-induced IL-6 expression was inhibited by 26.8% and 46.7%, respectively (Figure 3b). NHDF cells displayed consistent results with HaCaTs, where UVB-induced expression of MMP-1 and IL-6 was inhibited in the presence of ADSC-CM.

### 2.4. The Effects of ADSC-CM in MMP-1 and Procollagen Type I mRNA Expression in HaCaTs and NHDFs

**Figure 4:**
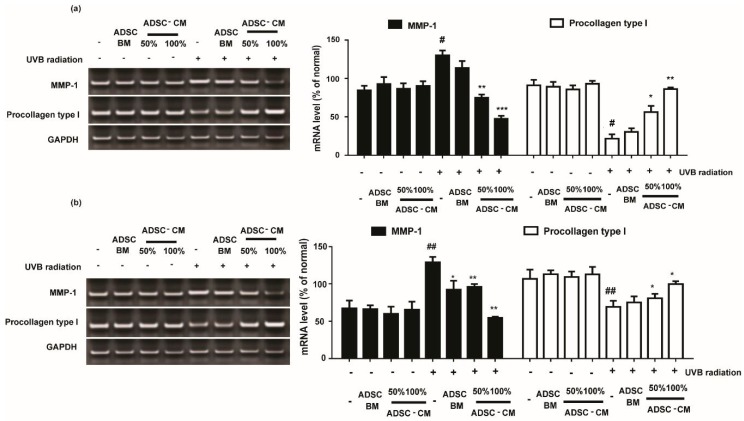
The effects of ADSC-CM on the mRNA expression of MMP-1 and procollagen type I in HaCaTs and NHDFs. HaCaTs (**a**) and NHDFs (**b**) were irradiated with UVB followed by treatment with 50% or 100% ADSC-CM for 24 h, respectively. All data are shown as the mean ± SD of at least three independent experiments. ^#^ *p* < 0.05 and ^##^ *p* < 0.01, compared with the non-irradiated control. * *p* < 0.05, ** *p* < 0.01, and *** *p* < 0.001, compared with the irradiated, ADSC-CM free control.

Cells treated with various concentrations of ADSC-CM resulted in downregulated UVB-induced MMP-1 expression. As shown in Figure 4, 100%ADSC-CM treatment reduced the MMP-1 expression by 63.5% and 57.9% in HaCaTs and NHDFs, respectively.

As the most abundant structural protein, procollagen type I is important for the synthesis of type I collagen. It was found that UVB irradiation significantly decreased the levels of procollagen type I expression. Following 100% ADSC-CM treatment, the mRNA expression of procollagen type I was gradually increased by 399.0% and 144.1% in HaCaTs and NHDFs, respectively (Figure 4).

### 2.5. ADSC-CM Treatment Alleviates the Phosphorylation of MAPK/AP-1

**Figure 5:**
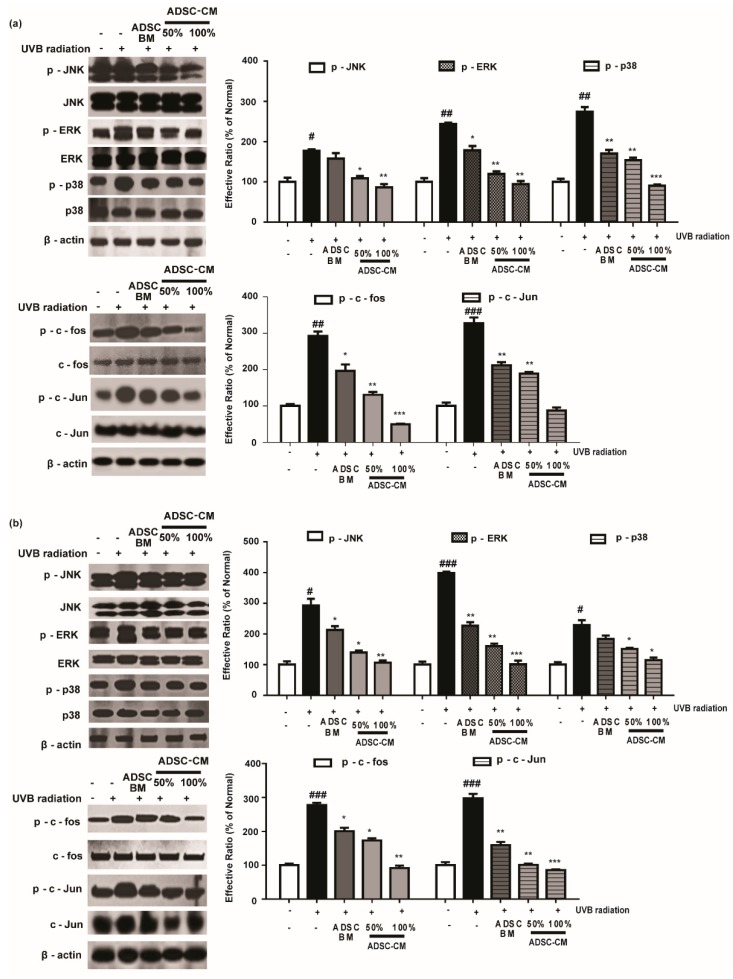
The effects of ADSC-CM on UVB-induced activation of MAPK/AP-1 signaling. HaCaTs (**a**) and NHDFs (**b**) were irradiated with UVB, followed by treatment with 50% or 100% ADSC-CM for 1 h and 4 h, respectively. The phosphorylation levels of ERK, JNK, p38, c-Jun, and c-Fos were measured by western blotting. The results are shown as the mean ± SD of at least three independent experiments. ^#^ *p* < 0.05, ^##^ *p* < 0.01, and ^###^ *p* < 0.001, compared with the non-irradiated control. * *p* < 0.05, ** *p* < 0.01, and *** *p* < 0.001, compared with the irradiated, ADSC-CM free control.

Since the MMP-1 promoter contains AP-1 binding sites, we assessed the phosphorylation rate of the MAPK/AP-1 signaling pathway to understand the mechanism of action of ADSC-CM. As shown in Figure 5, 100% ADSC-CM inhibited UVB-induced p-JNK, p-ERK, and p-p38 levels by 51.3%, 61.2%, and 67.2% in HaCaTs, respectively, while UVB-induced p-c-fos and p-c-Jun expression was inhibited by 83.2% and 73.2%, respectively. Similar results were found in NHDFs for the effects of ADSC-CM on UVB-induced MAPK/AP-1 phosphorylation (Figure 5).

### 2.6. ADSC-CM Attenuated NF-κB Expression and Downregulates the Activity of HO-1

**Figure 6:**
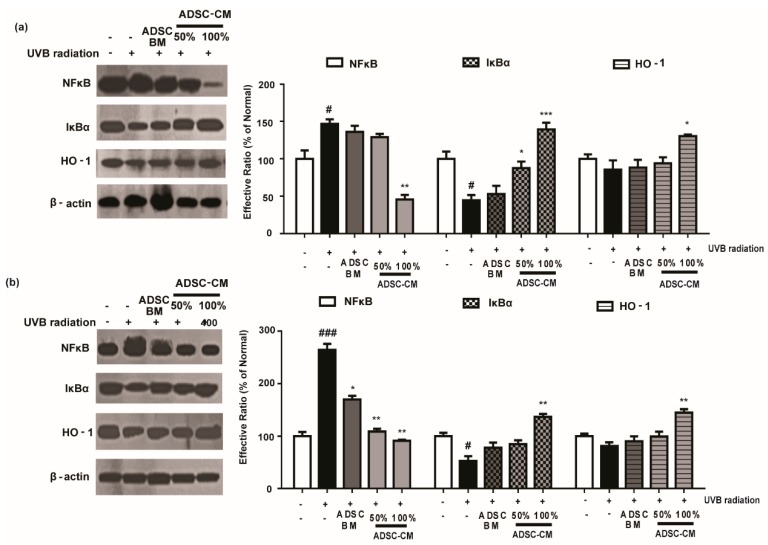
The effects of ADSC-CM on UVB-induced NF-κB/HO-1 activation in HaCaTs (**a**) and NHDFs (**b**). HaCaTs and NHDFs were irradiated with UVB, followed by treatment with 50% or 100% ADSC-CM for 2 h, respectively. The levels of NF-κB, IκBα, and HO-1 were measured using western blotting. The band intensities were quantified with densitometry and calculated as a percentage of the basal response. The results are shown as the mean ± SD of at least three independent experiments. ^#^ *p* < 0.05 and ^###^ *p* < 0.001, compared with the non-irradiated control. * *p* < 0.05, ** *p* < 0.01, and *** *p* < 0.001, compared with the irradiated, ADSC-CM free control.

To further investigate the mechanism of action of ADSC-CM, we examined the expression of NF-κB, IκBα, and HO-1 level. As shown in Figure 6, UVB exposure significantly increased NF-κB activity and downregulated the level of IκBα in HaCaT and NHDF cells. Notably, high-concentration ADSC-CM treatment decreased UVB-induced activation of NF-κB by 69.0% and 65.5% in HaCaT and NHDF cells, respectively (Figure 6). Moreover, ADSC-CM treatment downregulated UVB-induced activation of HO-1 (Figure 6).

### 2.7. ADSC-CM Treatment Has Regulatory Effects on TGF-β/Smad

**Figure 7:**
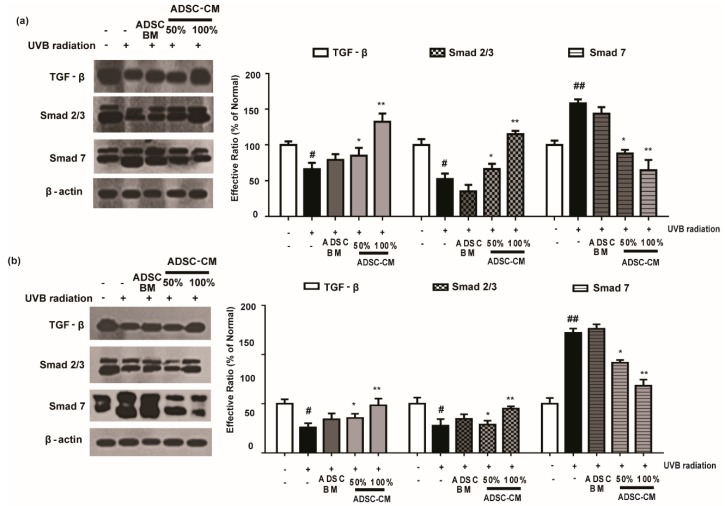
The effects of ADSC-CM on TGF-β, Smad2/3, and Smad7 expression in UVB-irradiated HaCaTs (**a**) and NHDFs (**b**). HaCaTs and NHDFs were irradiated with UVB, followed by treatment with 50% or 100% ADSC-CM for 1.5 h. The levels of TGF-β, Smad2/3, and Smad7 were detected by western blotting. The results are shown as the mean ± SD of at least three independent experiments. ^#^ *p* < 0.05 and ^##^ *p* < 0.01, compared with the non-irradiated control. * *p* < 0.05 and ** *p* < 0.01, compared with the irradiated, ADSC-CM free control.

As shown in Figure 7, UVB irradiation caused a decrease in the expression levels of TGF-β and Smad 2/3 proteins. Following the treatment with ADSC-CM, the expression of these proteins were increased in a dose-dependent manner. In particular, 100% ADSC-CM treatment recovered the expression of TGF-β and Smad to those in the non-irradiated control (Figure 7). It was found that UVB irradiation upregulated Smad 7 expression and that 100% ADSC-CM treatment reduced UVB-induced upregulation of Smad 7 by 59.2% in the HaCaT group. Similar results were found in NHDF cells for the photoprotective effects of ADSC-CM (Figure 7).

## 3. Discussion

The aim of the present study was to evaluate the potential of ADSC-CM to alleviate UVB-induced premature skin aging. We focused our attention on ADSCs because they can be manufactured with easy procedures with a greater proliferation rate than embryonic stem cells [14]. The multi-pluripotency and rapid proliferation of ADSCs facilitate their applications in skin regeneration [15]. ADSCs have been exploited in clinical studies for treating a variety of medical indications including sequelae of burns, radiodermatitis, sequelae of trauma. The wound-healing activity of ADSCs is mainly mediated by the activation of keratinocytes and dermal fibroblasts [16].

ADSCs secrete various growth factors that manage damaged neighboring cells, which has been identified as an essential function of ADSCs [17]. Indeed, the growth factors and chemokines in the supernatant provide a microenvironment for the stem cells to differentiate under culture conditions. Among the genes examined, basic fibroblast growth factor (bFGF), hepatocyte growth factor (HGF), and vascular endothelial growth factor (VEGF) facilitate endothelial cells to migrate and exert angiogenic effects on the wound transudate, which have been studied extensively for skin repair (Figure S1) [18,19]. In addition, MSC-derived extracellular vesicles can facilitate a wide range of therapeutic applications [20,21,22].

ADSC-CM is rich in secretory factors and has been shown to be capable of accelerating wound-healing in animal models by stimulating collagen synthesis in dermal fibroblasts [23]. In our previous study, it was observed that UVB-induced photoaging causes abnormality of primary skin fibroblasts and keratinocytes [24,25]. In the present study, we used these cells as models to evaluate the photoprotective effects of ADSC-CM and understand the underlying mechanisms for protecting UVB-induced oxidative stress.

HaCaTs and NHDFs are known to function as barriers to the “aging ray” UVB to prevent skin photoaging [26]. During UVB radiation, the system faces disturbance, and several cytokines and growth factors stimulate the transcription of MMPs. Increased MMP-1 protein expression will cause the degradation of collagen types I, II, and III. Collagen is an important extracellular component in human skin that undergoes continuous remodeling and turnover. Decrease in collagen synthesis and an excess of MMP-1 are thought to be the key features of the pathology of various inflammatory and skin aging diseases [27]. Our data indicate that ADSC-CM could prevent UVB-irradiated MMP-1 secretion and upregulate procollagen type I synthesis (Figure 3 and Figure 4), highlighting the potential of ADSC-CM for inhibiting UVB-induced wrinkle formation.

We further studied the mechanism of action of ADSC-CM. UVB-generated ROS (Figure 1) affects MAPK signaling cascades including JNK/MAPK, ERK1/2 MAPK, and p38 MAPK, and can activate the downstream effector protein AP-1 as well as NF-κB. In the present study, it was found that ADSC-CM could efficiently downregulate UVB-induced activation of the MAPK signaling pathway and transcriptionally downregulate proinflammatory target proteins such as IL-6. In addition, ADSC-CM can inhibit UVB-induced activation of AP-1 signaling transducers, which is associated with the induction of HO-1 and MMPs in human skin [26]. Moreover, ADSC-CM is shown to promote TGF-β, a key pro-fibrotic cytokine that is known to regulate cell proliferation, differentiation, and ECM production. Owing to its function for stimulating the migration of fibroblasts and collagen synthesis, ADSC-CM is under clinical investigation for treating skin aging [28]. Our results showed that UVB irradiation downregulates Smad 2/3 and upregulates Smad 7 in both HaCaT and NHDF cells. The ability of ADSC-CM to block the perturbation of TGF-β may be important for its photoprotective effects.

In summary, in the present study, we found that ADSC-CM could protect HaCaTs and NHDFs against UVB-induced photoaging by reducing the secretion of IL-6 and promoting the level of ROS through multiple pathways, leading to decreased MMP-1 production and increased procollagen type I expression. Our study shed light on the cosmetic or therapeutic applications of ADSC-CM for treating UVB-induced skin aging.

## 4. Materials and Methods

### 4.1. Chemicals

StemPro^®^ MSC SFM basal medium CTS (A10334-01), Dulbecco’s modified Eagle’s medium (DMEM), StemPro^®^ MSC SFM supplement CTS (A10333-01), fetal bovine serum (FBS), L-glutamine (25030-081), Gentamicin Reagent Solution (15710-064), DPBS CTS™ without magnesium, calcium (A12856), DPBS with calcium, magnesium (A12858), penicillin/streptomycin solution, TrypLE Select CTS (A12859), and Trizol were obtained from Gibco BRL (Grand Island, NY, USA). ELISA kits for bFGF, HGF, VEGF, MMP-1, and IL-6 were purchased from R&D Systems, Inc. (Minneapolis, MN, USA). Antibodies were obtained from Cell Science (Canton, MA, USA) and Santa Cruz Biotechnology (Santa Cruz, CA, USA).

### 4.2. ADSC Culture and Conditioned Medium Preparation

ADSCs were obtained from ThermoFisher Scientific (R778110, San Jose, CA, USA). In brief, ADSCs express the following cell-surface proteins including CD166, CD105, CD90, CD73, CD44, and CD29 (>95%) and are negative for CD45, CD31, CD14, and Lin1(<2%). ADSCs between passage 4 to 7 were used for the experiments. ADSCs cells were grown on CTS-coated T75 flasks and maintained in ADSC-SM (Table 1) at 36 °C–38 °C in an atmosphere containing 4–6% CO_2_. At 80%–100% confluency, ADSCs were washed with DPBS without calcium and magnesium and subcultured with pre-warmed TrypLe Select CTS for cell detachment. ADSCs were conditioned by exposure to ADSC-BM (Table 1) at 80–90% confluency for 24 h. After 24 h after treatment, ADSC-CM (Table 1) was centrifuged and then filtrated with 0.22 μm filters to remove cell debris. ADSC-CM was used at 50% and 100% of the medium volume for experiments and stored at −20 °C for further analyses.

### 4.3. Cell Culture and UVB Irradiation

HaCaTs and NHDFs were grown in DMEM supplemented with 10% heat-inactivated FBS and 1% penicillin-streptomycin at 37 °C. Cells were subjected to UVB irradiation at 125 mJ cm^−2^ for HaCaTs and 144 mJ cm^−2^ for NHDF by a Bio-Link BLX-312 (Vilber Lourmat GmbH, Paris, France) when 80%–90% confluency was achieved. HaCaTs were used between passage 10 to 40 for all experiments and NHDF cells were used between passage 2 to 10. Afterward, the cells were rinsed with PBS and instantly treated with conditioned medium.

### 4.4. Intracellular ROS Generation

To determine intracellular ROS levels, HaCaT cells were subjected to UVB (125 mJ/cm^2^), followed by treatment with different conditioned media for 2 h. After 24 h post treatment, the ADSC-CM-treated cells were stained with 30 μM DCFH-DA (Merck KGaA, Darmstadt, Germany) for 30 min at 37 °C in the dark. The cells were then analyzed by flow cytometry (FACS Accuri C6TM; Becton-Dickinson, San Jose, CA, USA).

### 4.5. Hoechst 33258 Staining

NHDFs were seeded onto sterile glass coverslips in 6-well plates at a density of 5 × 10^5^/well and grown to 80% confluency. After exposure to UVB at a density of 144 mJ cm^−2^, cells were administrated with ADSC-CM for 72 h. Afterward, cells were fixed with 4% paraformaldehyde for 15 min, rinsed twice with PBS and dyed with Hoechst 33258 solution for 10 min at room temperature. The morphology of apoptotic cells were recorded by a fluorescence microscope (400×, AMF4300, Life EVOS, Waltham, MA, USA). The apoptotic rate was calculated as a percentage of apoptotic cells over the total number of cells in five randomly selected areas.

### 4.6. MMP-1 and IL-6 Inhibition Assay

The activity of MMP-1 and IL-6 in the medium was determined using the ELISA kit following the manufacturer’s instructions. Each sample was analyzed in triplicate.

### 4.7. RNA Preparation and Reverse Transcription (RT)-PCR

Isolation of total RNA and the RT-PCR assay were performed at 24 h after ADSC-CM-treatment in a Veriti Thermal Cycler (Applied Biosystems, Foster City, CA, USA) as described [29]. GAPDH was used as the house keeping gene. Each experiment was repeated at least three times.

### 4.8. Western Blot Analysis

Cell lysates of HaCaTs and NHDFs were assayed as described [24,25]. The alteration of the MAPK, TGF-β, NF-κB, and AP-1 pathways were analyzed at 1, 1.5, 2, and 4 h after UVB irradiation, respectively. Each experiment was repeated at least three times.

### 4.9. Statistical Analysis

The data were expressed as means ± SD of three independent experiments. Statistical comparisons between different treatments were made using one-way analysis of variance followed by the Tukey test. Statistical significance was set at *p* < 0.05, *p* < 0.01, and *p* < 0.001 for the t-test.

## Figures and Tables

**Table 1:** Detailed components of the medium in this study.

Abbreviation	Name Refinement	Component (For 100 mL)
ADSC-BM	ADSC-basal medium	StemPro^®^ MSC SFM Basal Medium CTS™ (100 mL)
ADSC-SM	ADSC-serum supplemented medium	StemPro^®^ MSC SFM Basal Medium CTS™ (84 mL); StemPro^®^ MSC SFM Supplement CTS™ (15 mL); L-glutamine (2 mM); Gentamicin Reagent Solution (5 µg/mL final concentration)
ADSC-CM (100%)	ADSC-conditioned medium (100%)	Supernatants collected from ADSC culture (80–90% confluency)
ADSC-CM (50%)	ADSC-conditioned medium (50%)	The serum-free DMEM and ADSC-CM at ratio 1:1 (v:v)

## Supplementary Materials

Supplementary materials can be found at https://www.mdpi.com/1422-0067/21/1/49/s1.
