# Supplementary material for: Conditioned Medium from Human Adipose-Derived Mesenchymal Stem Cell Culture Prevents UVB-Induced Skin Aging in Human Keratinocytes and Dermal Fibroblasts
Source: Int J Mol Sci. 2019 Dec 19;21(1):49. doi: 10.3390/ijms21010049 (PMC6981944; doi:10.3390/ijms21010049)
Supplement: Supplementary file 1 [file ijms-21-00049-s001.pdf]

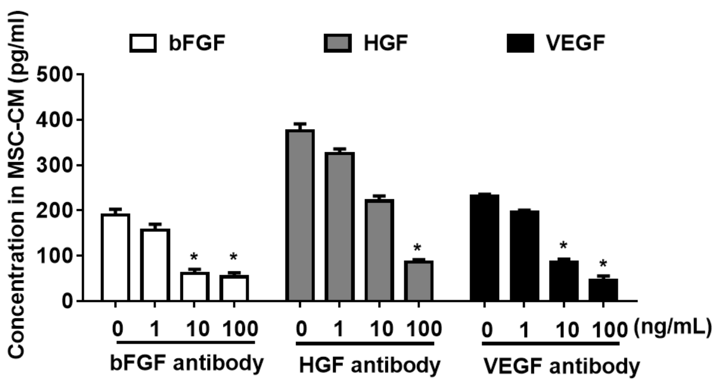

Figure S1. Growth factors in conditioned medium. bFGF, HGF and VEGF were determined via an enzyme-linked immunosorbent assay (ELISA) using commercially available ELISA sets. bFGF, fibroblast growth factor; VEGF, vascular endothelial growth factor; HGF, hepatocyte growth factor.* $p < 0.05$,* $p < 0.01$ and * $p < 0.001$, compared with control.
